# Nanoparticle-induced systemic toxicity and immune response in *Galleria mellonella* larvae

**DOI:** 10.3389/fphar.2025.1625472

**Published:** 2025-09-10

**Authors:** Kusal Shasheen Payoe, Kavita Gadar, Emmanuel Flahaut, Ronan R. McCarthy, Gudrun Stenbeck

**Affiliations:** ^1^ Centre for Genomic Engineering and Maintenance, Department of Biosciences, College of Health and Life Sciences, Brunel University London, Uxbridge, United Kingdom; ^2^ Antimicrobial Innovations Centre, Department of Biosciences, College of Health and Life Sciences, Brunel University London, Uxbridge, United Kingdom; ^3^ Centre Interuniversitaire de Recherche et d’Ingénierie des Matériaux, Université Toulouse 3 Paul Sabatier, Institut National Polytechnique de Toulouse, Centre National de la Recherche Scientifique (CNRS), Université de Toulouse, Toulouse, France

**Keywords:** nanoparticle uptake, *in vivo* toxicity, *Galleria mellonella*, infection, immunosupression, haemocytes

## Abstract

**Introduction:**

Nanotechnology is one of the most rapidly advancing scientific fields, offering innovative solutions in diverse areas such as medicine, agriculture, and materials science. However, concerns regarding the environmental and biological toxicity of nanomaterials continue to rise. It is thus essential to develop reliable, ethical, and cost-effective models to assess the *in vivo* toxicity of Nanoparticles (NPs). This study aims to evaluate the immunotoxicity and systemic effects of various inorganic nanoparticles using *Galleria mellonella* (GM) larvae as a non-mammalian *in vivo* model.

**Methods:**

GM larvae were exposed to different types of NPs, including starch-coated and anionic superparamagnetic iron oxide nanoparticles (SPIONs), double-walled carbon nanotubes (CNTs), and gold nanoparticles (GNPs). Flow cytometry was used to monitor haemocyte numbers, while larval survival assays assessed mortality. Histological analyses were conducted to detect CNT accumulation in tissues. The immunosuppressive effects of GNPs were assessed in GM larvae challenged with sub-lethal doses of *Pseudomonas aeruginosa* and *Acinetobacter baumannii*.

**Results:**

The results demonstrate NP retention in GM tissues and showed that surface and size properties of NPs significantly influenced their biological effects. Anionic SPIONs lacking a starch coating caused greater haemocyte depletion and higher mortality than their biocompatible coated counterparts. GNP toxicity was found to be size‐dependent, with particles between 60 and 100 nm producing the most severe haemocyte depletion, which was comparable to that obtained with the immune suppressant cyclophosphamide.

**Conclusion:**

Overall, this study supports the use of *GM* larvae as an effective model for nanoparticle toxicity screening and demonstrates the usefulness of this model in detecting both toxic and immunosuppressive properties of nanomaterials.

## Introduction

Due to their unique physicochemical properties, nanoparticles (NPs) with an overall size of between 1 and 100 nm in at least one dimension are now widely employed in industrial, biomedical and cosmetic applications, for example, as catalysts and fillers as well as nanoparticulate biomaterials for orthopaedic surgery, UV filters in sunscreens and food additives (Jia and Schüth, 2011; Stark et al., 2015; Bouwmeester et al., 2014). Novel biomedical uses include drug, vaccine and gene delivery and diagnostics and bioimaging (Mabrouk et al., 2021). *In vivo*, NPs show enhanced solubility and bioavailability, including the ability to cross the blood-brain barrier, to enter the pulmonary system and to undergo enhanced absorption through tight junctions (Kohane, 2007), making them ideal drug delivery vehicles where superior drug bioavailability as well as target-specificity is required (Patra et al., 2018; Eker et al., 2024).

Despite these favourable characteristics, NP toxicity is still a concern. Toxicity is commensurate to the chemical nature of the core material, the NPs’ size and shape (Liu et al., 2022) their ability to generate reactive oxygen species (ROS), disrupt cellular compartments and induce immune responses (Ngobili and Daniele, 2016; Pondman et al., 2023). Hence, standardised methods for biosafety evaluation of newly formulated NPs are urgently required (Drasler et al., 2017; Moya-Andérico et al., 2021). *In vitro* studies using cell lines have shown that toxicity in response to NP uptake is strongly dependent on cell type (Kroll et al., 2011). For example, identical concentrations of SPIONs induced significant cytotoxicity in neuronal, glial and lung cells, but minimal toxicity in all other tested cell types (Li et al., 2013; Wei et al., 2021). Therefore, *in vivo* experiments are essential when investigating the biological effects of NPs on the regulation of pH, ionic strength and chemical composition of circulating blood (Moore et al., 2000). Furthermore, NPs have been shown to interact with the immune system and can induce adverse effects, such as hypersensitivity reactions and inflammation (Pondman et al., 2023). Efforts have thus been made to shield NPs from interactions with the immune system. We have shown that binding of soluble complement factors to CNTs enhances their uptake in macrophages and minimises inflammatory cytokine production (Kouser et al., 2018; Pondman et al., 2015). However, difficulties to effectively replicate and fully capture the immune response in *in vitro* systems necessitates *in vivo* experiments using animal models (Drasler et al., 2017). Nevertheless, animal models have raised ethical concerns, are costly and not suitable for high throughput screening. In line with the 3Rs - Reduction, Refinement, and Replacement - the adoption of non-animal and invertebrate animal alternatives, where scientifically viable, is now becoming increasingly relevant. Invertebrate models, such as the fruit fly *Drosophila melanogaster* or the nematode *Caenorhabditis elegans,* are cost efficient and do not fall under restrictive ethical regulations, thus warranting their widespread use. To this end, the larvae of the invertebrate *Galleria Mellonella* (GM) (the greater wax moth), have been developed as an alternative and effective tool to study *in vivo* toxicity. Due to their rapid growth rate, large size, and short life cycle (Cutuli et al., 2019), they can be used to study pathogen virulence, host-pathogen interactions and measure efficacy of various antimicrobial, fungicidal and other agents, including NPs, at an early stage during drug development (Jander et al., 2000; Maslova et al., 2024; Maslova et al., 2020; Opris et al., 2025). Importantly, GM larvae can be maintained at 37 °C (Peleg Anton et al., 2009), thus allowing for nanotoxicity studies to take place at the physiological human body temperature, which is especially important when studying the effect of NPs on infections. Furthermore, the larvae possess a complex innate immune system, closely resembling that of mammals (Hernandez et al., 2019; Gallorini et al., 2024). The cells of the larval haemolymph (haemocytes) are classified into six different types (prohemocytes, plasmatocytes, granular cells, coagulocytes, spherulocytes and oenocytoids) (Tsai et al., 2016), while the humoral immune response consists of opsonisation by complement-like proteins, melanisation and synthesis of lysozyme and small antimicrobial peptides (Trevijano-Contador and Zaragoza, 2018). As such, the utility of this model for studying both immunological and microbiological aspects of host-pathogen interactions has been widely recognised (Menard et al., 2021). Thus, GM larvae can be a useful tool to evaluate the direct immunotoxicity and immunosuppressive activity of engineered NPs and their potential for complementing current immunosuppressive drugs.

Furthermore, GM larvae are a good model for infection with human pathogenic bacteria, such as *Pseudomonas aeruginosa* and *Acinetobacter baumannii*, both of which are leading causes of nosocomial infections in cystic fibrosis patients, burn victims, and other immunocompromised individuals (Jander et al., 2000; Maslova et al., 2020).

In this study, we determined the *in vivo* biological safety of a range of nanoparticles, both with and without treatment with the immunosuppressive drug cyclophosphamide. We utilised flow cytometry as an accurate and reliable method for the quantification of total circulating haemocytes and employed cryo-sectioning and histochemistry to determine the systemic effects of injected NPs in GM larvae. Larval survival was studied with Kaplan Meier survival curves and reactive oxygen generation *in vivo* was assessed by measuring the concentration of 4-hydroxynonenal (4-HNE), a secondary product of lipoperoxidation (Poli and Schaur, 2000). Challenging the larvae with sub-lethal doses of highly virulent strains of *P. aeruginosa* and *A. baumannii* enabled us to study early immune responses to invasion of these two clinically important pathogens after immune suppression with cyclophosphamide and treatment with GNPs.

Our findings highlight the value of GM larvae in the study of the innate immune response to injected NPs and its usefulness for high-throughput investigation of NP immunotoxicity. Additionally, we discuss potential clinical applications of the tested nanoparticles to directly modulate the innate immune response, with the potential to complement current immunosuppressive therapies.

## Materials and methods

### Nanoparticle synthesis and characterisation

Acute *in vivo* toxicity of three different types of nanoparticles was assessed: superparamagnetic iron oxide nanoparticles (SPIONs), double walled carbon nanotubes (CNTs) and gold nanoparticles (GNPs).

SPIONs (nano-screenMAG) with an overall dimension of 100 nm were obtained from Chemicell, Berlin, Germany. The SPIONs were synthesised by Chemicell (Berlin, Germany) using a proprietary protocol converting acidic iron(II/III) salt into iron(II/III) carbonate, followed by successive thermal reactions to produce multidomain nanocrystals that were purified through magnetic separation (Schlenk et al., 2017). The SPIONs consist of a magnetite core covered with a lipophilic fluorescence dye and a second layer of a hydrophilic polymer. Two types of nano-screenMAG were used, starch coated (SC), with no overall charge, a PDi of 0.3 and zeta potential of 0–5 mV, and anionic (An) SPIONs, with a negative charge, a PDi of 0.3 and zeta potential of −15 mV (Chemicell, Berlin, Germany).

Three different CNTs, as characterised and described previously (Kouser et al., 2018; Pondman et al., 2014), were utilised: unmodified, oxidised (Ox) and Carboxymethyl cellulose (CMC) coated double walled carbon nanotubes a few micrometres long. The CNTs were synthesised by catalytic chemical vapor deposition of a mixture of CH_4_ and H_2_ at 1,000 °C on a Co:Mo MgO-based catalyst and subsequent treatment with an aqueous HCl solution to remove oxides and non-protected residual catalyst nanoparticles (Flahaut et al., 2003). After washing on a filtration membrane (polypropylene, 0.45 µm), these CNTs have a zeta potential of 0.4 mV (Bortolamiol et al., 2014). Briefly, for non-covalent functionalisation, wet CNTs were added to a solution of carboxymethyl cellulose (CMC) in PBS in a 1:1 ratio. Agglomerates were removed by centrifugation and excess CMC was removed by vacuum filtration (Kouser et al., 2018). For oxidised CNTs, a 1:1 w/v ration of wet CNTs and 3 M HNO_3_ (1 mg of dry equivalent of DWNTs per mL) was placed in an ultrasonic bath for 30 min and refluxed at 130 °C for 24 h as described by Bortolamiol et al. After cooling to room temperature, the solution was washed and filtered (polypropylene, 0.45 µm) (Bortolamiol et al., 2014). The ox-CNTs have a zeta potential of −40 mV (Bortolamiol et al., 2014).

Citrate capped GNPs of varying dimensions were obtained from BBI solutions, Kent, United Kingdom (20 nm and 60 nm GNPs) and Sigma-Aldrich, Poole, United Kingdom (100 nm GNPs). All GNPs were prepared using modified proprietary citrate reduction protocols (www.bbisolutions.com and www.cytodiagnostics.com). The 20 nm GNPs have a Z-average of 23.33 d.nm, a PDi of 0.069 and a zeta potential of −33.9 ± 5 mV. The 60 nm GNPs have a Z-average of 61.77, a PDi of 0.14 and zeta potential of −50.8 ± 12.9 mV. The 100 nm GNPs have a core size of 100 nm and a hydrodynamic size of 113 nm with a PDi of 0.04, and a zeta potential of −23 mV.

### Nanoparticle suspension and working concentrations

All NPs were suspended in PBS and dispersed in a water bath sonicator for 1 h before injections. Working concentrations were selected based on previously published *in vivo* toxicology studies.

#### CNTs

Deng et al. (2007) reported no adverse effects following injection of functionalised multiwalled CNTs (600 nm in length) at doses up to 24 mg/kg in male KunMing mice (Deng et al., 2007). Based on this, CNTs were administered at a dose of 10 mg/kg in this study.

#### SPIONs

Chertok et al. (2008) used 100 nm SPIONs at a concentration of 12 mg/kg for magnetic field-guided delivery to gliomas in male Fisher 344 rats whereas Prabhu et al. (2015) showed that a single injection of up to 350 mg/kg in BALB/c Swiss Albino mice was non-toxic (Chertok et al., 2008; Prabhu et al., 2015). Accordingly, SPIONs were injected into larvae at 15 mg/kg.

#### GNPs

Sonavane et al. (2008) observed no toxicity 24 h after injecting male ddY mice with various sizes of GNPs at concentrations as high as 1 g/kg (Sonavane et al., 2008). However, Cho et al. (2009) reported acute liver inflammation and apoptosis in mice at a dose of 4.26 mg/kg using 13 nm PEGylated GNPs (Cho et al., 2009). Based on these findings, GNPs were administered at a concentration of 5.6 mg/kg in this study.

### GM *in vivo* nanoparticle toxicity

#### Sorting GM

Commercially available *Galleria Mellonella* larvae (Lepidoptera: pyralidae, the greater wax moth) were acquired from LiveFood UK Ltd. (Somerset, United Kingdom). Only sixth instar larvae of indetermined sex (sexually dimorphic characteristics only emerge during the pupal stage (Campbell et al., 2024), were used for experiments, which do not require feeding and weigh approximately 200 mg. Prior to use, larvae were sorted to discard any damaged, already deceased, or pupated larvae and stored at 4 °C until use.

#### GM inoculations

Groups of 10 GM larvae were injected using a 22s-gauge microlitre syringe (Hamilton, Reno, NV, USA) in the right or left-hand side of the first set of prolegs. On day one, larvae were inoculated with either 10 μL PBS (Fisher Scientific, Loughborough, UK) (control group) or with one high dose of 10 μL 11 mM cyclophosphamide (CTX) (Acros organics, Fisher Scientific, Loughborough, UK) (used at a final concentration of 147 mg/kg). Larvae were incubated at 37 °C for 24 h. On day two, larval groups were inoculated with either 10 μL PBS (control) or 10 μL of the relevant test NPs. Larvae were monitored at 37 °C over the course of 72 h post injection. Mortality was determined by complete melanisation of the larval epicuticle and complete loss of motility (McCarthy et al., 2017). Mortality was initially assessed each hour for a total of 3 h, to measure acute NP toxicity. Thereafter, larval mortality was assessed in hourly steps to acquire larval survival numbers at 24, 48 and 72 h. *In vivo* NP toxicity assays were carried out as three independent experiments for each NP variant with n > 45 GM larvae per test. Statistically significant differences between larval survival were determined using the log-rank test (Prism 8.0, GraphPad Software, San Diego, CA, USA).

#### GM inoculations with P*seudomonas aeruginosa* and *Acinetobacter baumannii* bacterial strains

GM bacterial assays were carried out with two gram-negative hyper-virulent bacterial strains, *P. aeruginosa* PA14 and *A. baumannii* AB5075 in conjunction with the immunosuppressant cyclophosphamide or 60 nm GNPs. Respective larvae were injected in the first set of prolegs, with 10 μL PBS (Fisher Scientific, Loughborough, UK), 10 μL cyclophosphamide at a concentration of 0.0318 mg/10 μL (final concentration of 147 mg/kg) per larvae or 10 μL of 60 nm GNPs (final concentration of 5.6 mg/kg) and incubated at 37 °C for 24 h. After 24 h, larval groups were inoculated with 10 μL PBS (control) or 10 μL of serially diluted PA14 or AB5075 cultures. Mortality was assessed each hour for a total of 3 h, to measure acute NP toxicity. Thereafter, larval mortality was assessed in hourly steps to acquire larval survival numbers at 24, 48 and 72 h. Mortality was determined as before. Larval haemocyte quantification through flow cytometric analysis was done in triplicate for the respective larval test conditions in three independent NP toxicity assays. Similarly, *in vivo* toxicity of NPs within the differing larval physiological conditions, were carried out as three independent toxicity experiments.

#### Preparation of bacterial culture

To prepare the bacterial culture for subsequent GM inoculations, 5 mL of Lysogeny broth (LB) medium (Sigma-Aldrich, Poole, UK) was inoculated with PA14 or AB5075 and the bacteria was left to grow overnight at 37 °C. Thereafter, the bacteria were washed 3 times with PBS before injecting a concentration of 3 × 10^4^ CFU per larva for AB5075 and 22 CFU per larva for PA14. Larvae were then incubated at 37 °C and monitored over the course of 24 h.

#### Nanoparticle distribution (*in vivo*)

To qualitatively analyse haemocyte proliferation/immunotoxicity and for the visualisation of haemocyte-mediated NP internalisation, the larval haemolymph was extracted 24 h post-NP inoculation. Haemolymph from three larvae was pooled and immediately fixed with 4% phosphate-buffered paraformaldehyde pH 7 (PFA) to prevent cellular coagulation and haemolymph oxidation. Extracted haemocytes were stained with HOECHST 33342 at 10 nM for the visualisation of cellular nuclei, and AlexaFluor546 labelled Wheat Germ Agglutinin (WGA) for the visualisation of the plasma membrane, as per manufacturer’s guidelines (ThermoFisher, UK). Haemocytes were imaged with a DM400 fluorescent microscope (Leica, Wetzlar, Germany) using LAS software using the appropriate fluorescent channels and brightfield imaging. Confocal images were acquired with a Nikon Eclipse TE2000-S confocal microscope with 60x oil lens.

#### GM haemocyte quantification

**FIGURE 1:**
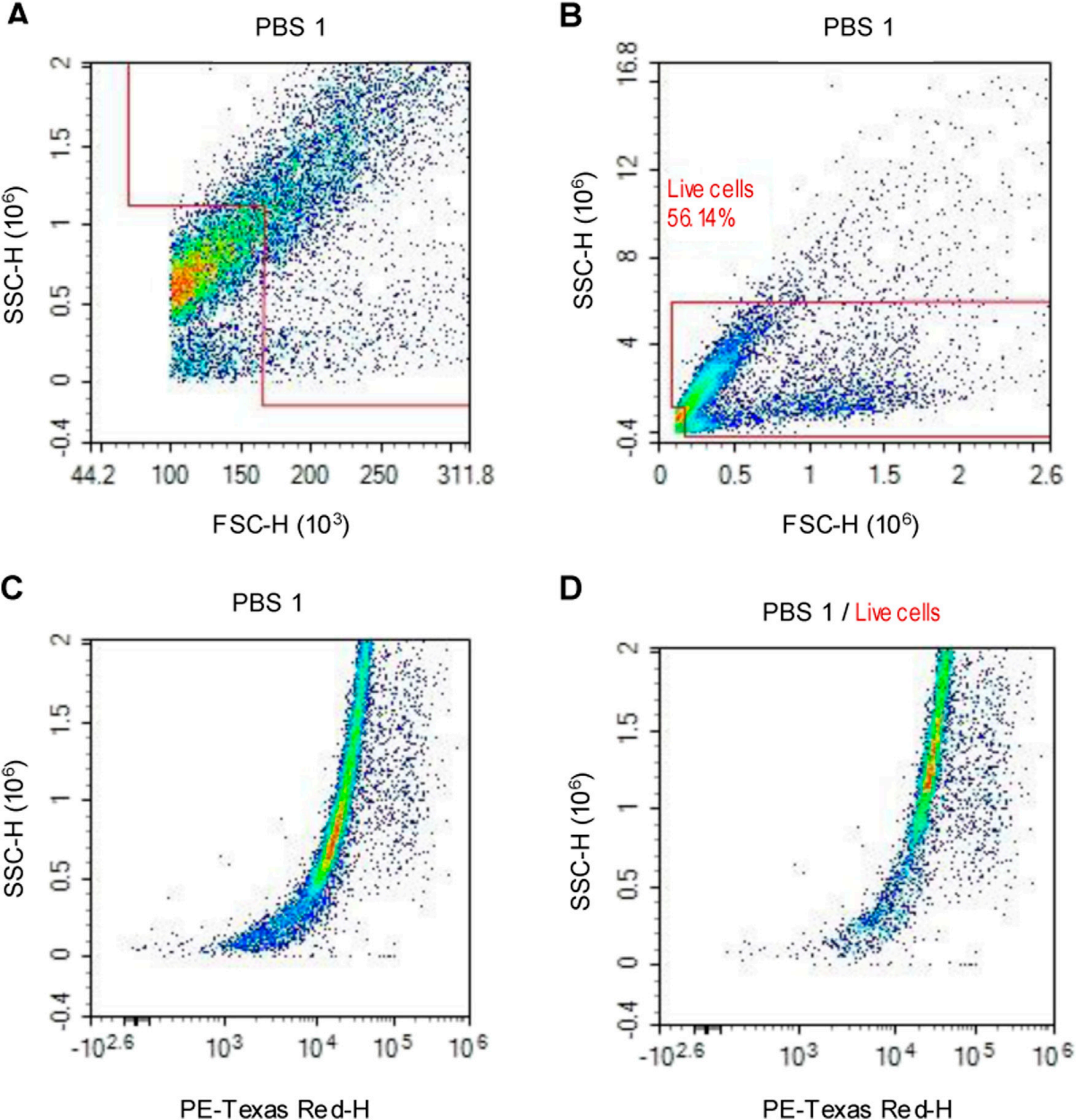
Flow cytometry density plot charts, representing the events/cells gathered from PBS control larvae. **(A)** Cellular debris is eliminated by the forward scatter/side scatter gating method. **(B)** In addition, aggregated cells are identified due to their high side scatter and are eliminated to only obtain individual cells. **(C)** All events/cells that are positive for the nuclear acid stain (PI) are visualised. **(D)** The polygon gate is applied to the PI positive events to confirm cellular debris elimination.

For flow cytometry, the haemolymph of three larvae from each experimental group was extracted, pooled and immediately fixed by adding 350 μL of 4% PFA to prevent oxidative discolouration and coagulation. Samples were incubated for 20–30 min on ice. To isolate haemocytes, samples were centrifuged at 4 °C for 5 min at 3,000×g. After washing the pelleted cells with PBS, cells were resuspended in 500 µL of PBS containing 2 ng/μL propidium iodide (PI) (Sigma-Aldrich, Poole, UK). The stain was used as secondary confirmation of debris removal during flow cytometry analysis, Figure 1. Samples were analysed on a NovoCyte flow cytometer (Agilent technologies, Santa Clara, United States).

The number of events/cells for each experimental group was acquired via polygon gating of the subsequent density plot charts, which display the axes of forward scatter height and side scatter height, Figure 1. Total haemocyte count (THC) per µl of the sample was calculated for each experimental group to determine haemocyte concentration. Cellular concentration was calculated by dividing the number of viable cells by the volume of sample analysed.

### Histological analysis

Groups of larvae were inoculated with PBS for the negative control or the appropriate test CNTs as stated above. Larvae were then incubated at 37 °C for 24 h. Larvae were fixed inside 15 mL falcon tubes to avoid significant body bending (2% PFA overnight at 4 °C). Larvae were then rinsed in PBS and euthanised by freezing at −20 °C for approximately 30 min. Thereafter, frozen larvae were embedded in OCT embedding compound (Fisher Scientific, Loughborough, UK) at −27 °C for 30 min. OCT blocks were sectioned at 20 µm on a Leica CM1860 Cryostat (Leica, Wetzlar, Germany) and the resulting GM cryosections were collected on snowcoat microscope slides (Leica, Wetzlar, Germany). The sections were stained with haematoxylin and eosin (H&E) per standard protocols and analysed using a Cytation 5 automated imaging microscope (Agilent technologies, Inc., Santa Clara, USA). Low and higher magnification images were acquired using an ×4 and ×10 PL FL phase objective, respectively. Scoring of the images was done in duplicate with one researcher blinded to which sections were obtained from CNT injected larvae.

### *In vivo* measurement of NP induced ROS production

For the quantification of NP induced cellular Reactive Oxygen Species (ROS, GM larvae were injected with the test NPs as described above. Injected larvae were incubated for 24 h at 37 °C. Thereafter, larval haemolymph was extracted and placed in Eppendorf tubes containing acid citrate dextrose (ACD) solution (Sigma-Aldrich, Poole, UK) as anticoagulant solution at a final concentration of 10% of extracted haemolymph volume and kept on ice. Haemolymph samples were centrifuged at 2,800 rpm for 5 min at 4 °C, to pellet the cells. The supernatants (larval plasma) were removed, aliquoted into Eppendorf tubes, snap frozen using liquid nitrogen and stored at −80 °C.

The levels of 4-hydroxynonenal (4-HNE) were measured using a 4-HNE ELISA kit (cat. no. E-EL-0128; Elabscience Biotechnology, Co., Ltd.), according to manufacturer’s instructions and normalised to total protein content. Protein concentration of larval plasma was determined with a micro-Bradford protein assay (Bio-Rad, London, UK) according to the manufacturer’s instructions using BSA as standard curve. Plasma samples from larvae injected with PBS were used as negative control and plasma from larvae injected with 10% hydrogen peroxide were used as positive control. The 4-HNE assay was developed according to the manufacturer’s instructions and optical density (OD) values were acquired using a Bio-Rad microplate reader (Bio-Rad, London, UK) at emission wavelength of 450 nm. 4-HNE levels were determined using a 4-HNE standard curve and displayed as ng/mL after normalisation to protein content.

### Statistical analysis

Data was generated from a minimum of 10 larvae per condition with two biological replicates per experiment. Power calculations assumed that between 40%–60% of larvae would die. A minimum of three independent experiments was carried out before data was pooled and averaged. All experiments included vehicle treated controls, which were used for comparison. Statistical tests were performed using Prism 8.0, GraphPad Software, San Diego, CA, USA. For survival curves, percentage of survival was compared to control group using the Log Rank (Mantel-Cox) statistical test to determine significance. For THC, data is presented as mean count/μl haemolymph ±SD. Standard unpaired T-tests with Welch’s correction was used, when comparing independent datasets. One-way ANOVA followed by Tukey’s multiple comparisons test was used to compare independent dataset across multiple groups. Differences between independent datasets were deemed to be of significance when P-value <0.05.

## Results

### Cyclophosphamide exerts immunosuppressive effects in *G. Mellonella* larvae

**FIGURE 2:**
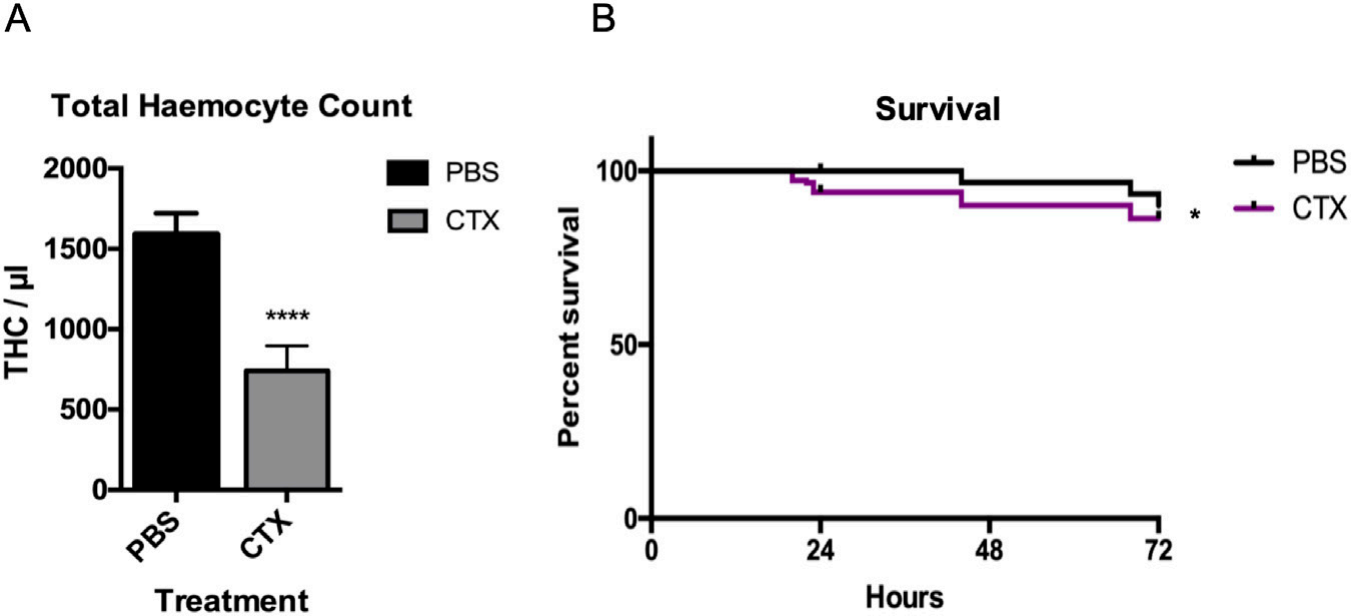
CTX induced immunosuppression in GM-larvae. GM larvae were injected with 147 mg/kg of the immunocytotoxic drug CTX. **(A)** Haemolymph was extracted after 24 h to numerate total circulating haemocyte counts (THC) by flow cytometry. THC was reduced by 46% compared to PBS controls (n = 3 independent experiments). Error bars show standard deviation. Asterixis represent statistically significant differences in THC in unpaired t-test (∗∗∗∗: p-value <0.0001). **(B)** GM larval survival was assessed over 72 h. CTX treatment induced a mild (4%) but significant (p < 0.05) toxic effect on the larvae (n = 3 independent experiments with >45 larvae per condition). Asterisks represent a statistically significant difference in larval survival, when compared to the relevant control, in a Log-Rank (Mantel- Cox) test (∗: p-value <0.05).

To investigate the possibility to induce immunosuppression in GM larvae, we injected larvae with 147 mg/kg of the immunocytotoxic drug cyclophosphamide (CTX). The CTX concentration was based on those used in animal studies to obtain immune suppression (Huyan et al., 2011). Haemolymph was extracted after 24 h to numerate total circulating haemocyte counts (THC) by flow cytometry. GM larval survival was assessed over 72 h (Figure 2). CTX treatment induced a mild (4%) but significant (p < 0.5) toxic effect on the larvae (Figure 2B), whereas a strong suppression of haemocyte numbers was observed (−46%, n = 3 independent experiments, p < 0.001) (Figure 2A). These results demonstrate that CTX can be used in GM larvae to induce immune suppression at concentrations comparable to those used in rodent models.

### Systemic *in vivo* toxicity of selected nanoparticles in *G. Mellonells larvae*

**FIGURE 3:**
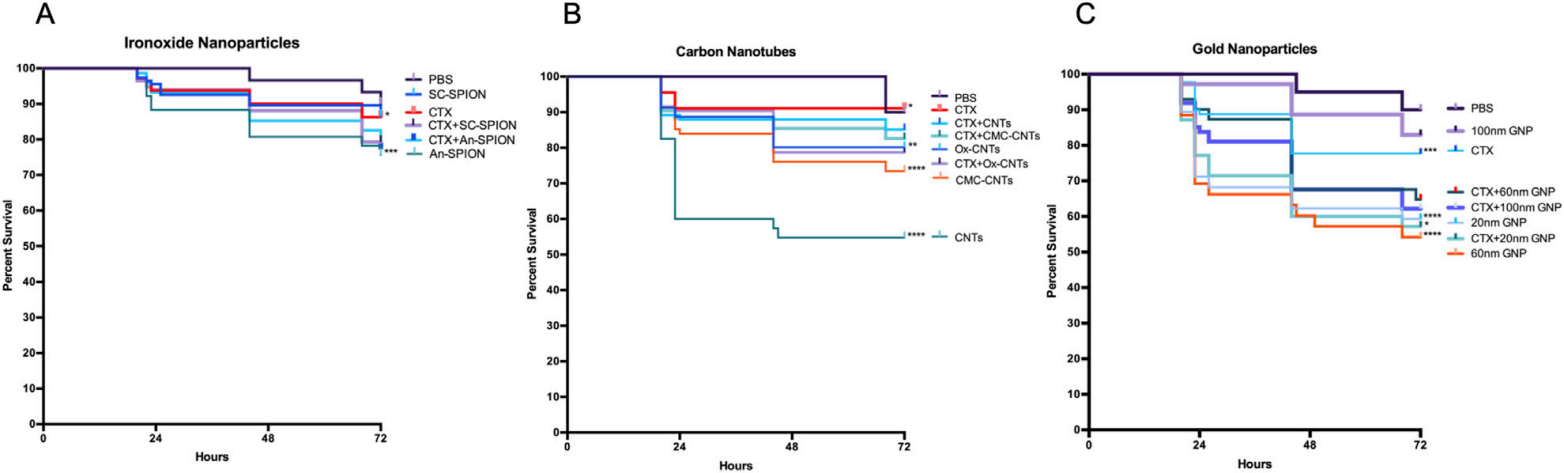
Larval survival analysis to determine *in vivo* toxicity of NPs. Kaplan-Meier survival curves, presenting percentage survival of GM larvae over 72 h after injection of NPs, n ≥ 45 larvae per condition with n = 3 independent experiments. Asterisks represent a statistically significant difference in larval survival, when compared to the relevant control, in a Log-Rank (Mantel- Cox) test (∗∗: p-value <0.01; ∗∗∗∗: p-value <0.0001). **(A)** Survival of larvae injected with 15 mg/kg SPIONs (starch-coated and anionic charged) was assessed in controls (PBS injected) and immunosuppressed (CTX-treated) larvae. **(B)** Percentage survival of GM larvae after injection with 10 mg/kg CNTs (oxidised, unmodified and CMC-coated) in controls (PBS injected) and immunosuppressed (CTX treated). **(C)** Percentage survival of GM larvae after injection with 5.6 mg/kg GNPs of differing sizes (20, 60 and 100 nm) in controls (PBS injected) and immunosuppressed (CTX-treated) larvae.

Different synthetic nanoparticles were used to study their systemic toxicity and immune toxicity in comparison to CTX. NP concentrations were chosen to be in line with published *in vivo* toxicology studies (Deng et al., 2007; Chertok et al., 2008; Sonavane et al., 2008). Based on these studies, commercially available SPIONs and GNPs were injected at concentrations of 15 mg/kg (SPIONs), and 5.6 mg/kg (GNPs). CNTs were used at 10 mg/kg. GM larval survival was assessed over 72 h. As shown in Figure 3, Kaplan Meier survival curves demonstrate varying levels of systemic toxicity, which is dependent on NP size, composition, and immunological state of the larvae. For SPIONs, the survival curves show that starch coated (SC) SPIONs induce limited toxicity, with no significant larval death observed in the control and post-immune suppression with CTX (3.4%–12% increase in larval death compared to control, Figure 3A). In contrast, An-SPIONs caused significant larval mortality in the control group (16% above baseline), which was slightly reduced following CTX-induced immunosuppression (12.8% above baseline, Figure 3A).

All CNTs induced significant larval death, with unmodified CNTs being the most toxic and oxidised CNTs the least (39% and 11% respectively). However, after immunosuppression with CTX, no significant larval death was observed for all tested CNTs (Figure 3B). GNPs, on the other hand, induced significant larval death in the controls with the exception of the 100 nm size GNPs (Figure 3C). Post-immunosuppression with CTX, an additive negative effect of CTX and GNP injection was observed for the 100 nm GNPs (from 7.8% to 31%) whereas for the smaller size GNPs this effect was variable, resulting in no effect (20 nm) or a reduction in toxicity (from 36% to 25.2%) for the 60 nm GNPs (Figure 3C).

### Cyclophosphamide treatment increases immunotoxicity of charged gold- and iron nanoparticles in *G. Mellonella* larvae

NPs strongly interact with both the innate and adaptive immune systems (Engin and Hayes, 2018). Direct immunotoxicity results in immune suppression (Ngobili and Daniele, 2016). To determine the effect of injected NPs on GM larvae’s innate immune system, haemocytes were isolated 24 h after treatment to measure changes in THC by flow cytometry.

**FIGURE 4:**
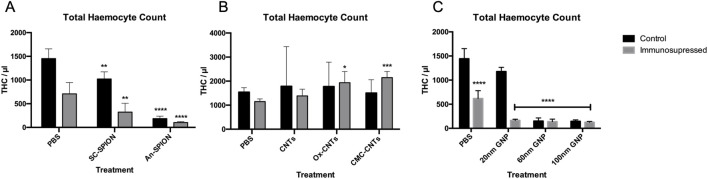
Flow cytometric analysis of isolated larval haemocytes to determine Total Haemocyte Count. Graphs presenting mean GM larval THC content per microlitre +/− SD for **(A)** SPIONs, **(B)** CNTs and **(C)** GNPs, in both the control and after treatment with CTX for 24 h prior to injection of NPs. THC was measured via flow cytometry analysis 24 h post-NP injections. Controls were injected with only PBS. Asterisks represent a statistically significant difference when compared to the relevant control, in an unpaired t-test (∗: p-value <0.05; ∗∗: p-value <0.01; ∗∗∗: p-value <0.001; ∗∗∗∗: p-value <0.0001). Results from n = 3 independent experiments are shown. Flow cytometric analysis was carried out on duplicate samples from each sample.

All SPIONs induced a significant decrease in THC, which was exacerbated post-treatment with CTX (Figure 4A). In contrast, CNTs (oxidised, unmodified and CMC coated) did not induce a significant change in THC. However, differently to what was observed with the SPIONs, pre-treatment of GM larvae with CTX had no influence on the increase in THC count after injection with oxidised and CMC-CNTs, with both treatments inducing a statistically significant increase in THC (Figure 4B). All GNPs induced significant decreases in THC. This effect was synergistic with the CTX treatment since further decreases in THC were observed in the 20, 60 and 100 nm GNPs treated samples (Figure 4C). Treatment with the 60 and 100 nm GNPs alone reduced THC to a level comparable to that observed with CTX treatment.

### Nanoparticles induce reactive oxygen species production *in vivo* in *G. Mellonella* larvae

**FIGURE 5:**
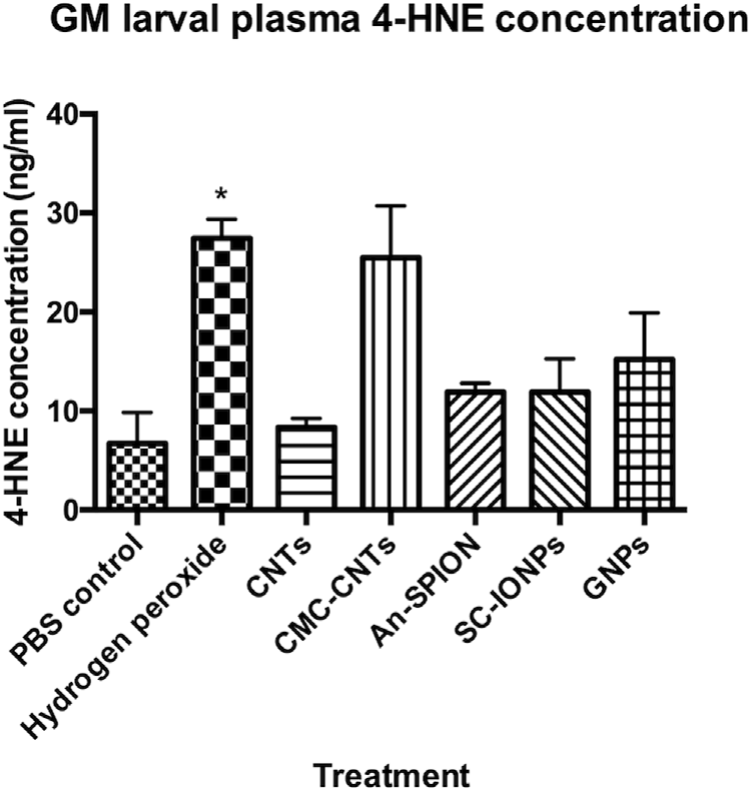
4-HNE-ELISA measuring generation of ROS *in vivo*. Graph presenting larval plasma 4-HNE concentrations (ng/mL), measured in plasma samples acquired from groups of larvae, 24 h post-larval injections with either SC-SPIONs or An-SPION, 100 nm GNPs, CNTs and CMC-CNTs. The figure presents mean concentration ±SD of two independent experiments, assayed in duplicate and normalised to protein content. Asterisks represent statistically significant difference when compared to the PBS control, in an unpaired t-test (∗: p-value <0.05)

NP immunotoxicity/immunomodulation has been demonstrated in multiple studies, with the most common mechanism being the induction of oxidative stress due to the excessive generation of intracellular ROS and subsequent activation of inflammatory responses (Wang Y.-L. et al., 2024). To quantify NP-induced bursts in cellular ROS production *in vivo*, we conducted a 4-HNE ELISA using isolated larval plasma samples. Plasma samples from larvae treated with hydrogen peroxide were used as positive control. Samples from larvae injected with SC-SPIONs, An-SPIONS, 100 nm GNPs and CMC-CNTs, showed an increase in 4-HNE concentration when compared to the negative control (Figure 5). However, under these experimental conditions, only plasma samples from larvae treated with hydrogen peroxide (positive control) yielded statistically significant results. These findings indicate that the NPs only induce a mild increase in tissue ROS.

### *In vivo* uptake of selected nanoparticles into *G. Mellonella* haemocytes

**FIGURE 6:**
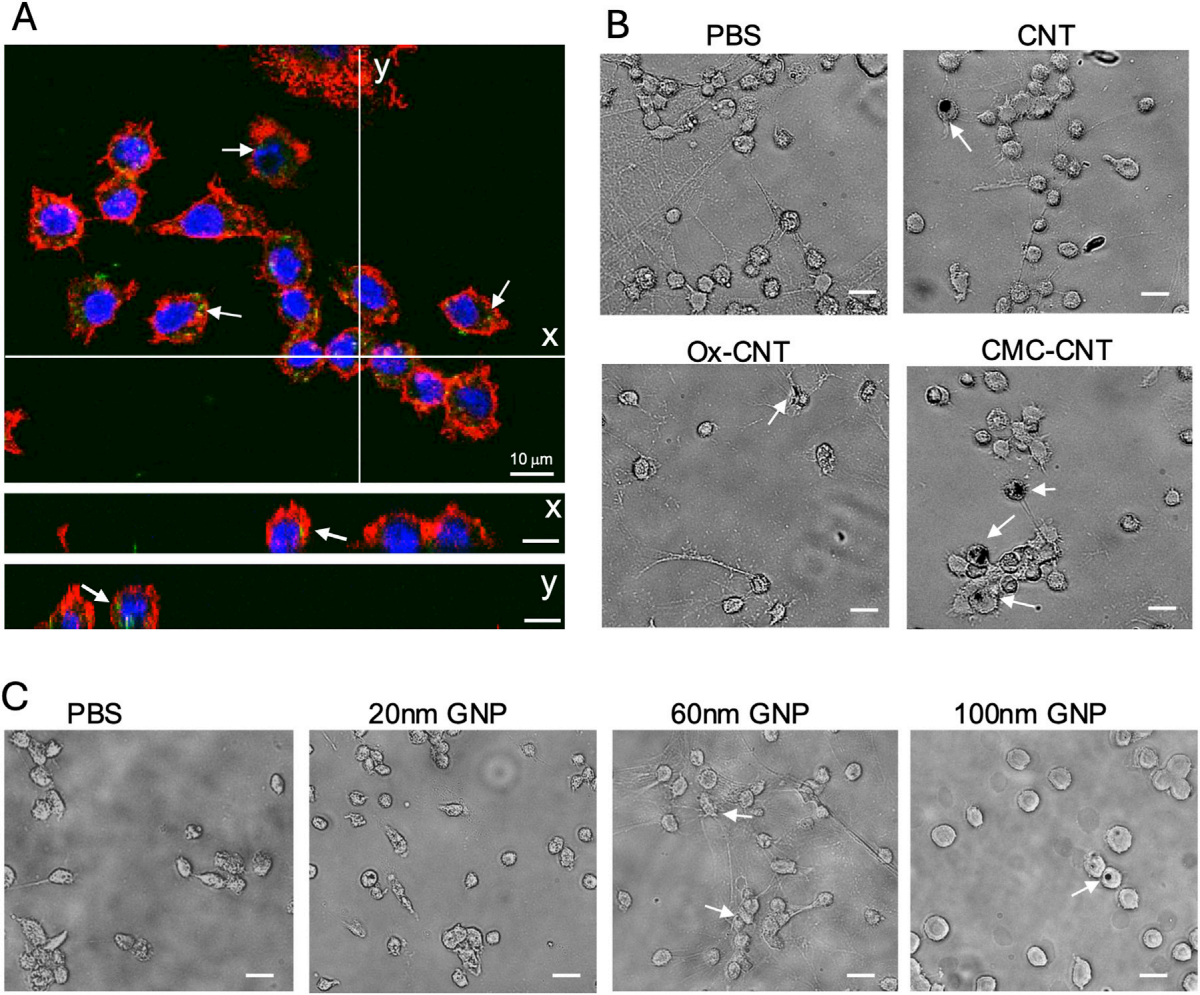
Uptake of NPs into GM haemocytes 24 h post-injection. Haemocytes isolated from plasma samples from larvae injected with 15 mg/kg SC-SPION were fixed and stained with AlexaFluor546-labeled WGA to reveal the plasma membrane (red). The nucleus was stained with Hoechst 33342 (blue). **(A)** single confocal section is shown, scale bars 10 μm. (y and x) orthogonal views of the same confocal image taken at the lines indicated in **(A)** demonstrate uptake of fluorescent SC-SPION (green, arrows). **(B)** Brightfield images of haemocytes isolated from larvae treated with 10 mg/kg CNT show altered morphology compared to control haemocytes and are clustered around CNTs (dark spots, arrows). **(C)** Brightfield images of haemocytes isolated from GNP injected larvae (5.6 mg/kg) have similar morphology to control haemocytes. Only larger size GNPs can be seen as dark spots associated with the cells (arrows; scale bar 15 μm). Representative images of n = 3 independent experiments with three biological replicates per experiment are shown.

To assess the effect of NP exposure on the GM immune system, haemocytes were isolated 24 h after NP injection and either assessed with confocal microscopy to image cellular uptake of fluorescent SPIONs or brightfield imaging for GNPs and CNTs. Single confocal sections show cellular uptake of fluorescent Sc-SPIONs in GM haemocytes treated with 15 mg/kg SC-SPION. The SC-SPIONs were distributed throughout the cytoplasm and accumulated in the perinuclear region (Figure 6A). A similar distribution was observed for the An-SPIONs (data not shown). Brightfield images of haemocytes isolated from larvae treated with 10 mg/kg CNTs showed altered morphology compared to control haemocytes. In addition, haemocytes from CNT-treated larvae were clustered around CNTs (Figure 6B). In contrast, brightfield images of haemocytes isolated from GNP injected larvae (5.6 mg/kg) show similar morphology to control haemocytes (Figure 6C). Only larger size GNPs can be seen as dark spots associated with the cells.

### Differential tissue accumulation of carbon nanotubes in *G. Mellonella* larvae

**FIGURE 7:**
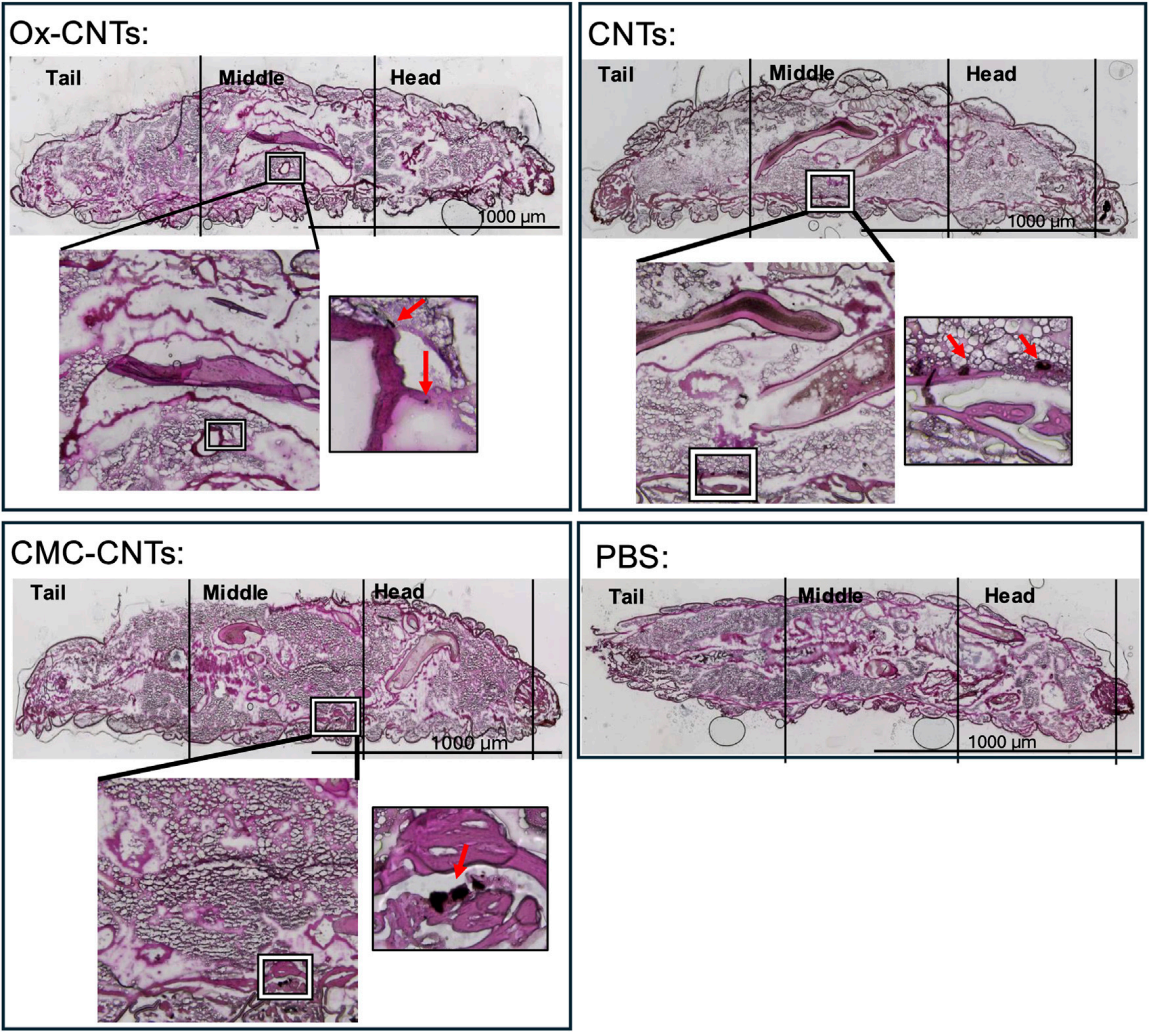
Histochemical analysis of larval cryosections showing *in vivo* CNT accumulation. Histological analysis of CNT distribution 24 h post-injection in GM larvae. H&E stain of 20 μm cryosections along the rostro-caudal axis taken 24 h after injection of 10 mg/kg CNTs. Larvae sections present tail, middle and head. ×4 magnification overview photomicrographs of the injected larva are shown in the coloured bright field (CBF) channel (scale bars 1,000 μm) where the aggregated CNTs are identifiable by their relative darkness (indicated by square). For comparison, an overview photomicrograph of a larva injected with vehicle (PBS) is shown. The zoomed in overlay of the CBF shows ×10 magnification photomicrograph detailing CNT localisation indicated in the square.

CNTs have been shown to significantly accumulate in animal tissues (Aoki and Saito, 2020). To assess CNT distribution in GM larvae, frozen cryosections were taken along the larval rostro caudal (RC) axis and stained with H&E to detect CNT localisation. CNTs are easily distinguishable from the surrounding tissue due to their relative darkness under bright field (Figure 7).

At 24 h post-injection, larvae inoculated with PBS displayed a healthy anatomical phenotype with no noticeable changes in organ localisation and anatomical shape (Figure 7), as previously reported (Kristensen, 2003). In contrast, oxidised CNTs were localised in small aggregates located in close proximity to the Malpighian tubules. CMC-CNTs were localised in larger aggregates within striate muscle fascicles of the larval prolegs. Similarly, unmodified CNTs localised in large aggregates in close proximity to the striate muscle fascicles.

In summary, CNTs are mainly associated with the outer wall of the digestive tract, muscle fascicles and the Malpighian tubules, whilst the lumen of the digestive tract appears to be clear of any CNT deposits.

### Immunosuppression in *G. Mellonella* larvae differentially alters responses to bacterial infection with *Pseudomonas aeruginosa* and *Acinetobacter Baumannii*

**FIGURE 8:**
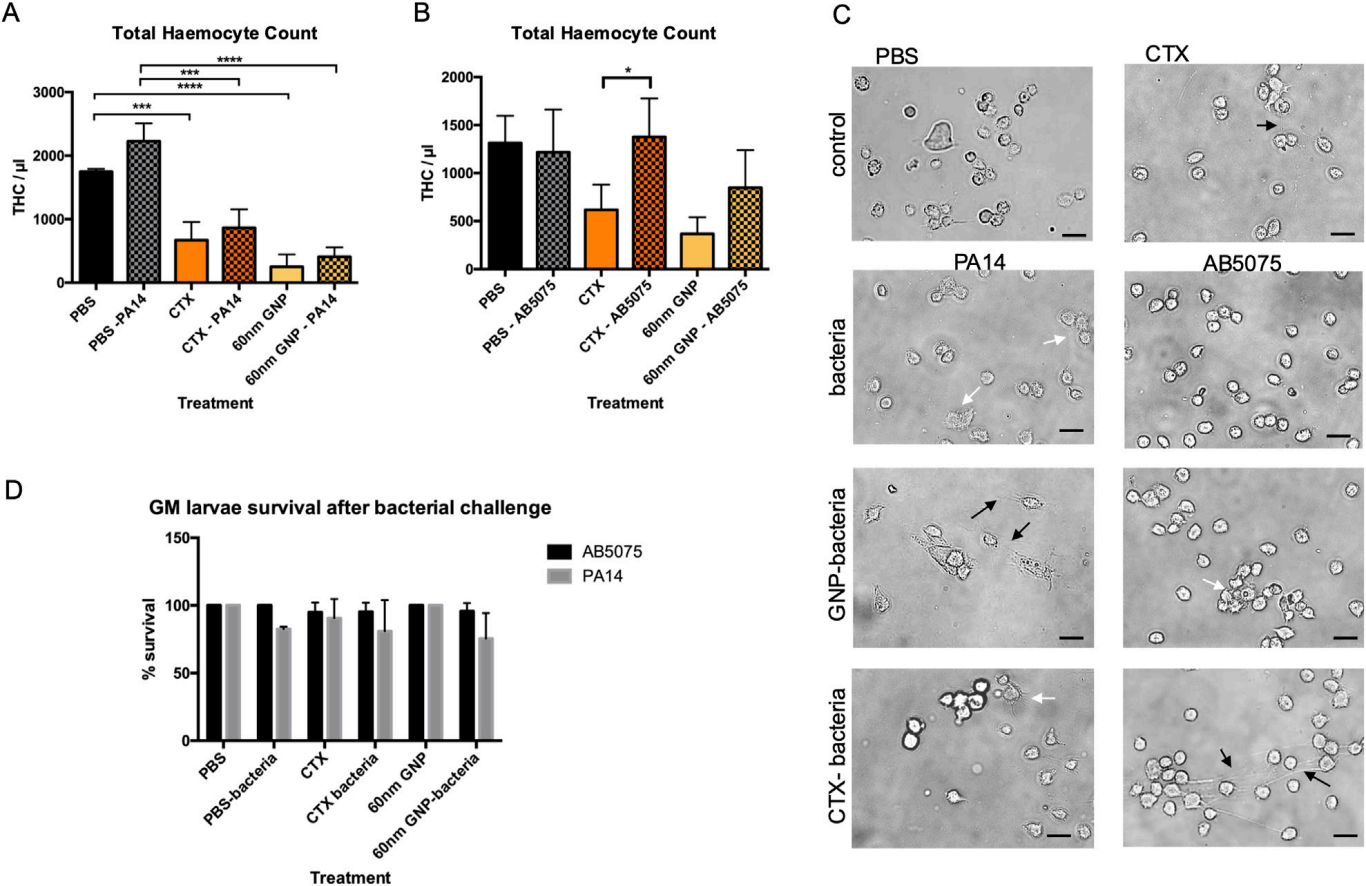
Flow cytometric survival and morphological analysis of isolated larval haemocytes to determine THC and survival after immune challenge. **(A)** Graph representing changes to GM larval THC, induced by CTX, *P Aeruginosa* PA14 and 60 nm GNP treatments either alone or in combination. **(B)** Graph representing changes to GM larval THC, induced by CTX, *A Baumannii* AB5075 and 60 nm GNP treatments either alone or in combination. THC was measured via flow cytometry analysis, 24 h post-bacterial inoculations in duplicates. As controls, THC of larvae injected with only PBS, CTX or 60 nm GNPs was measured. Mean THC from three independent experiments +/-SD with three biological replicates are shown. Asterisks represent a statistically significant difference when compared to the relevant control, in an unpaired t-test (∗:p-value <0.05; ∗∗:p-value <0.01; ∗∗∗:p-value <0.001; ∗∗∗∗:p-value <0.0001). **(C)** Representative Brightfield images of larval haemocytes taken from the same experiments show haemocyte morphology. Scale bars 15 μm. Changes to haemocyte morphology are indicated with white and black arrows. White arrows point to cells with increase appearance of filopodia and clustering. Black arrows point to extracellular material permeating from the haemocytes. **(D)** Survival of GM larvae treated with CTX, CTX, *P Aeruginosa* PA14, *A Baumannii* AB5075 and 60 nm GNPs either alone or in combination was analysed by determining percentage of live/dead larvae 24 h after treatment, n = 3 independent experiments with >45 larvae per condition, error bars show standard deviation.

Challenging GM larvae with the hyper-virulent *P. aeruginosa* (PA14) or *Acinetobacter Baumannii* strain (AB5075) reference strains (Figure 8) induced a significant increase in THC compared to the PBS control. This effect was supressed by preincubation with CTX for 24 h in PA14-treated samples (Figure 8A), whereas in AB5075-injected larvae, the THC count stayed elevated despite immunosuppression (Figure 8B). Pre-treatment with 60 nm GNPs for 24 h induced a highly significant decrease in THC with a pronounced suppression of the THC increase in response to PA14 but not to AB5075 (Figures 8A,B).

For PA14-exposed larvae, immunosuppression translated into an increase in larval death, and a similar trend was observed after pre-treatment with 60 nm GNPs. AB5075-injected larvae did not die to a significant extent and were protected from immunosuppression-elicited death (Figure 8D). Analysis of haemocyte morphology (Figure 8C) revealed changes to the appearance of the cells extracted from the treated larvae. While haemocytes from larvae treated with PA14 appeared enlarged with increased levels of filopodia, the cells extracted from AB5075 treated larvae were small and round. Pre-treatment with 60 nm GNPs enhanced appearance of extracellular material permeating from haemocytes in PA14, but not in AB5075 treated larvae whereas pre-treatment with CTX reduced cell aggregation in PA14 samples, but induced appearance of extracellular material in AB5075 samples, which could also be observed in haemocytes extracted from larvae treated only with CTX.

## Discussion

With the rapid expansion of nanotechnology, efficient and accurate toxicity assays are essential for the use of NPs in the clinic. Although rodent models provide valuable insights, they are costly and limited by ethical concerns. In this study, *GM* larvae were used as a non-rodent, *in vivo* alternative to assess nanotoxicity under immune suppression. We demonstrated here the usefulness of the GM model to study NP toxicity and responses to nosocomial infection under immunosuppression. CTX, one of the most used agents in cancer chemotherapy and for the treatment of immunological disorders, robustly reduces THC in GM larvae within 24 h with only mild overall toxicity (Figure 2B).

Due to the similarities between the innate immune cellular components in GM larvae and mammals, larval THC was used as an indicator of NP immunotoxicity/immunosuppression. Significant levels of immunotoxicity were observed for both SPION variants (Figure 4A); however, it was more significant for An-SPIONs, both in control and CTX-treated larvae and translated into significant larval death 72 h post-inoculation (Figure 3A). This difference in toxicity is most likely due to the lack of biocompatible coating in An-SPIONs, as various surface modifications of magnetite NPs are known to increase their biocompatibility (Singh et al., 2010). Furthermore, the immunotoxic effect could be due to increased levels of cellular oxidative stress, alterations of the cytoskeleton and genotoxicity in response to NP uptake (Singh et al., 2010). Fluorescent microscopy imaging shows that GM larval haemocytes avidly internalise NPs (Figure 6A) with internalised SPIONs localised throughout the cytoplasm and in close proximity to the cell nucleus. Interestingly, data reviewed by Shah and Dobrovolskaia indicates that iron oxide nanoparticle accumulation in rat alveolar macrophages and RAW264.7 macrophage like cells induced widespread reprogramming of genes involved in oxidative stress and inflammation (Shah and Dobrovolskaia, 2018). However, Stroh et al. showed that oxidative stress is a transient event, linked to free iron concentration in the cells, which does not lead to long-term cytotoxicity (Stroh et al., 2004).

To investigate and measure cellular ROS production in the larvae, we conducted an *in vivo* assay to quantify cellular ROS in response to NP exposure and/or cellular uptake (Zhang et al., 2011). A 4-HNE-lipid peroxidation ELISA was used for the first time in conjunction with GM larval biological samples. This assay showed that at the used concentrations, SPIONs only lead to limited ROS generation 24 h post-treatment (Figure 5). Our results are thus in line with the *in vitro* data obtained by Stroh et al. (2004). However, our results clearly demonstrate a negative effect on larval haemocytes that is exacerbated by CTX, which could be due to increased SPION uptake in CTX-treated larvae. This suggests that cellular responses, including endoplasmic reticulum stress, mitochondrial damage, and autophagy are affecting haemocyte survival (Park et al., 2014). An increase in ROS production similar to treatment with SPIONs was observed in larvae injected with 100 nm GNPs (Figure 5). The 100 nm GNPs also induced a comparable effect to the An-SPIONs on THC and larval survival, reflecting the lack of biocompatible coating and the overall negative charge of these NPs (Figures 3C, 4C). In general, GNPs showed a size-dependent effect on THC, with both 60 nm and 100 nm GNPs inducing a strong THC depletion. This immunotoxic effect has also been observed *in vitro* with human peripheral blood lymphocytes and murine splenic lymphocytes, which were significantly inhibited by GNPs at a concentration of 200 mg/kg (Devanabanda et al., 2016).

While significant decreases in larval THC were observed across all tested GNPs, only the 20 and 60 nm GNPs induced significant larval death, whereas the 100 nm variant showed only mild systemic toxicity (Figure 3C). This is in line with the study by Vecchio et al. that assessed toxicity of citrate capped GNPs of different sizes (5, 15, 40 and 80 nm) in *D. melanogaster* (Vecchio et al., 2012). While the flies were ingesting the GNPs with their food, the results clearly demonstrate a size dependent toxicity with a strong reduction of *Drosophila* lifespan, while a concentration dependent toxicity was observed for the presence of DNA fragmentation and changes in expression of genes linked to stress response, DNA damage and apoptosis (Vecchio et al., 2012). Furthermore, Isoda et al. assessed toxicity in mice treated with 10, 50 and 100 nm GNPs and their results showed that 10 and 50 nm GNPs cause kidney damage, while the 100 nm GNPs did not cause any systemic effect (Isoda et al., 2020). In our experiments, all GNP variants showed enhanced systemic toxicity after immunosuppression with CTX (Figure 3C). However, the effect varied and was highest for the 100 nm GNP. This could be due to the fact that these GNPs are more efficiently removed in immune competent larvae whereas in immunosuppressed larvae, the lack of immune cell uptake and systemic removal could induce greater levels of systemic toxicity.

The highest level of 4-HNE was observed in the CMC-CNT treated samples, with 4-HNE concentrations comparable to the positive control. Conversely, in the unmodified CNTs 4-HNE increases were negligeable. This data is in line with a study by Hsieh and Jafvert demonstrating that coated single-wall CNTs greatly enhance the rate of superoxide formation, which is not simply based on charge, but most likely due to the increased ability of the coated CNTs to scavenge ROS and in turn to generate greater quantities of more stable ROS (Hsieh and Jafvert, 2015). Furthermore, CMC-CNTs were more avidly taken up by the larval haemocytes than the other variants (Figure 5B), increasing the likelihood of cellular ROS production in an effort to clear them from the cells (Qi et al., 2024). Additionally, Meunier et al. have shown that phagocytosis of unmodified double-walled CNTs by human monocytes is necessary to induce IL-1β secretion, which stimulates a strong inflammatory response (Meunier et al., 2012). This inflammatory response is higher in CNTs with reduced solubility (Dumortier et al., 2006) and most likely explains the lack of THC reduction that we observed in larvae treated with the CNT variants (Figure 4B). Interestingly, CTX pre-treatment did not suppress THC (Figure 4B), indicating that there was a strong activation of the larval innate immune system by all tested CNT variants leading to a systemic inflammatory response. Despite a lack of effect on THC in CNT-treated larvae, CTX pre-treatment reduced larval death, an effect that was more pronounced in larvae treated with the unmodified CNTs (from 54% to 15%), whereas for both CMC-CNT and ox-CNTs, this protective effect was more limited. These results highlight the importance of CNT dispersion not only on larval death (27% CMC-CNTs and 20% ox-CNTs compared to 54.7% for the unmodified CNTs), but also on the innate immune response (Figure 4B). Furthermore, an increased clearance of CNTs by haemocytes could contribute to the increased larval survival in CTX-treated larvae. In mammals, CTX was shown to enhance the population of monocytes undergoing active endocytosis, thus leading to enhanced clearance by liver and spleen of IgG-sensitized erythrocytes (Ziccheddu et al., 2013; Palermo et al., 1991; Giordano and Isturiz, 1983).

In addition to innate immune-mediated inflammatory responses, the observed systemic toxicity (Figure 3B) could also be due to the *in vivo* accumulation of CNTs, contributing to larval death. Histochemistry is commonly carried out on paraffin embedded whole body sections of GM larvae to investigate host-pathogen interactions (Perdoni et al., 2014), progression of pathogen infection *in vivo* (Djainal et al., 2020) and pathogen invasiveness with cryo-imaging (Sheehan et al., 2018). In this study, we utilised for the first time histological analysis on GM larval cryo-sections stained with H&E, which show CNT aggregates localised in close association with the digestive tract, striate muscle fascicles and the Malpighian tubules (Figure 7).

To measure the effect of immunosuppression caused by NPs in the context of bacterial infection, we used 60 nm GNPs. These NPs, which are close in size to those used in commercial lateral flow assays, showed robust immunosuppression (Kim et al., 2016). GM larvae are highly susceptible to *P*. *aeruginosa* PA14 infections whereas higher CFU are tolerated for *A. baumannii* AB5075 (Tsai et al., 2016). When the GM larval innate immune system was challenged with these nosocomial infection causing bacteria, a significant increase in larval THC was observed (Figure 8A). For *P*. *aeruginosa*, this increase was not observed after CTX- or 60 nm GNP-induced immunosuppression whereas *A. baumannii* induced THC increases even after immune suppression (Figure 8A). This result highlights similarities to human infection and findings in rodent models, where *A. baumannii* triggers a strong increase in cytokine release (Wang H. et al., 2024). *P*. *aeruginosa* infections on the other hand show a strong induction of neutrophil recruitment to the site of infection with extensive formation of neutrophil extracellular traps (NETs) (Kamoshida et al., 2015). Interestingly, we saw an increase in extracellular material resembling the NETs observed in mammalian host surrounding haemocytes isolated from PA14 treated larvae especially after pre-treatment with 60 nm GNPs (Figure 8C). At the tested concentrations, reduction in larval survival after immunosuppression was only observed in *P. aeruginosa* infected larvae, which, however, did not reach statistical significance (Figure 8B). It is important to note that we used sublethal bacterial doses for both bacteria species, which allowed us to investigate the immediate early effects of infection (Ambrosi et al., 2020), including THC count.

NP-induced immunosuppression through direct immune interaction is often overlooked. Metal oxide NPs, like SPIONs, show immunosuppressive and anti-inflammatory properties - e.g., IONPs reducing humoral immune responses (Shah and Dobrovolskaia, 2018). Noble metal NPs, such as GNPs, interact with both innate and adaptive immune components, but their immunosuppressive mechanisms remain underexplored (Ngobili and Daniele, 2016; Wang Y.-L. et al., 2024). Depending on the context, NP-induced immunosuppression can be beneficial (e.g., enhancing drug efficacy, treating autoimmune diseases, improving transplant tolerance) or harmful (e.g., reducing host defences against infections or cancer) (Pondman et al., 2023). It is thus important to evaluate the immunosuppressive effect of novel NP formulations. Our results confirm the importance of NP coating to reduce toxicity. However, they also demonstrate that not all coatings have the anticipated effect as further physical features, such as dispersibility and tissue uptake, are important factors to take into consideration when developing novel nanotherapeutics. While our study sheds light on the immediate early effects of NP immunotoxicity, more work needs to be done to elucidate the molecular mechanisms leading to the observed immunosuppression elicited by GNPs and SPIONs and the long-term effects of immunosuppression in the larvae. Future work could include a quantification of the NP retention in the larvae including development defects.

In summary, our study shows that GM larvae provide a valuable intermediate model to assay NP *in vivo* toxicity, enabling more accurate predictions of their behaviour in whole organisms and their immunomodulating potential.

## Data Availability

The original contributions presented in the study are included in the article/supplementary material, further inquiries can be directed to the corresponding author.

## References

[B1] Ambrosi C. Scribano D. Sarshar M. Zagaglia C. Singer B. B. Palamara A. T. (2020). Acinetobacter baumannii targets human carcinoembryonic antigen-related cell adhesion molecules (CEACAMs) for invasion of pneumocytes. mSystems 5, e00604-20. 10.1128/msystems.00604-20 33361319 PMC7762790

[B2] Aoki K. Saito N. (2020). Biocompatibility and carcinogenicity of carbon nanotubes as biomaterials. Nanomaterials 10, 264. 10.3390/nano10020264 32033249 PMC7075247

[B3] Bortolamiol T. Lukanov P. Galibert A.-M. Soula B. Lonchambon P. Datas L. (2014). Double-walled carbon nanotubes: quantitative purification assessment, balance between purification and degradation and solution filling as an evidence of opening. Carbon 78, 79–90. 10.1016/j.carbon.2014.06.051

[B4] Bouwmeester H. Brandhoff P. Marvin H. J. P. Weigel S. Peters R. J. (2014). State of the safety assessment and current use of nanomaterials in food and food production. Trends Food Sci. and Technol. 40, 200–210. 10.1016/j.tifs.2014.08.009

[B5] Campbell J. S. Pearce J. C. Bebes A. Pradhan A. Yuecel R. Brown A. J. P. (2024). Characterising phagocytes and measuring phagocytosis from live Galleria mellonella larvae. Virulence 15, 2313413. 10.1080/21505594.2024.2313413 38357909 PMC10877982

[B6] Chertok B. Moffat B. A. David A. E. Yu F. Bergemann C. Ross B. D. (2008). Iron oxide nanoparticles as a drug delivery vehicle for MRI monitored magnetic targeting of brain tumors. Biomaterials 29, 487–496. 10.1016/j.biomaterials.2007.08.050 17964647 PMC2761681

[B7] Cho W.-S. Cho M. Jeong J. Choi M. Cho H. Y. Han B. S. (2009). Acute toxicity and pharmacokinetics of 13 nm-sized PEG-coated gold nanoparticles. Toxicol. Appl. Pharmacol. 236, 16–24. 10.1016/j.taap.2008.12.023 19162059

[B8] Cutuli M. A. Petronio petronio G. Vergalito F. Magnifico I. Pietrangelo L. Venditti N. (2019). Galleria mellonella as a consolidated *in vivo* model hosts: New developments in antibacterial strategies and novel drug testing. Virulence 10, 527–541. 10.1080/21505594.2019.1621649 31142220 PMC6550544

[B9] Deng X. Jia G. Wang H. Sun H. Wang X. Yang S. (2007). Translocation and fate of multi-walled carbon nanotubes *in vivo* . Carbon 45, 1419–1424. 10.1016/j.carbon.2007.03.035

[B10] Devanabanda M. Abdul L. S. Madduri R. (2016). Immunotoxic effects of gold and silver nanoparticles: inhibition of mitogen-induced proliferative responses and viability of human and murine lymphocytes *in vitro* . J. Immunotoxicol. 13, 897–902. 10.1080/1547691X.2016.1234522 27754724

[B68] Djainal W. A. S. Shahin K. Metselaar M. Adams A. Desbois A. P. (2020). Larva of greater wax moth *Galleria mellonella* is a suitable alternative host for the fish pathogen Francisella noatunensis subsp. orientalis. BMC Microbiol. 20, 8. 31918661 10.1186/s12866-020-1695-0 PMC6953311

[B11] Drasler B. Sayre P. Steinhäuser K. G. Petri-Fink A. Rothen-Rutishauser B. (2017). *In vitro* approaches to assess the hazard of nanomaterials. NanoImpact 8, 99–116. 10.1016/j.impact.2017.08.002

[B12] Dumortier H. Lacotte S. Pastorin G. Marega R. Wu W. Bonifazi D. (2006). Functionalized carbon nanotubes are non-cytotoxic and preserve the functionality of primary immune cells. Nano Lett. 6, 1522–1528. 10.1021/nl061160x 16834443

[B13] Eker F. Duman H. Akdasci E. Bolat E. Sarıtaş S. Karav S. (2024). A comprehensive review of nanoparticles: from Classification to application and toxicity. Molecules 29, 3482. 10.3390/molecules29153482 39124888 PMC11314082

[B14] Engin A. B. Hayes A. W. (2018). The impact of immunotoxicity in evaluation of the nanomaterials safety. Toxicol. Res. Appl. 2, 2397847318755579. 10.1177/2397847318755579

[B15] Flahaut E. Bacsa R. Peigney A. Laurent C. (2003). Gram-scale CCVD synthesis of double-walled carbon nanotubes. Chem. Commun. (Camb), 1442–1443. 10.1039/b301514a 12841282

[B16] Gallorini M. Marinacci B. Pellegrini B. Cataldi A. Dindo M. L. Carradori S. (2024). Immunophenotyping of hemocytes from infected Galleria mellonella larvae as an innovative tool for immune profiling, infection studies and drug screening. Sci. Rep. 14, 759. 10.1038/s41598-024-51316-z 38191588 PMC10774281

[B17] Giordano M. Isturiz M. A. (1983). Enhancement of erythrophagocytosis by cyclophosphamide. Cell. Immunol. 81, 187–191. 10.1016/0008-8749(83)90225-3 6616625

[B18] Hernandez R. J. Hesse E. Dowling A. J. Coyle N. M. Feil E. J. Gaze W. H. (2019). Using the wax moth larva Galleria mellonella infection model to detect emerging bacterial pathogens. PeerJ 6, e6150. 10.7717/peerj.6150 30631644 PMC6322482

[B19] Hsieh H.-S. Jafvert C. T. (2015). Reactive oxygen species generation and dispersant-dependent electron transfer through single-walled carbon nanotubes in water. Carbon 89, 361–371. 10.1016/j.carbon.2015.03.052

[B20] Huyan X.-H. Lin Y.-P. Gao T. Chen R. Y. Fan Y. M. (2011). Immunosuppressive effect of cyclophosphamide on white blood cells and lymphocyte subpopulations from peripheral blood of Balb/c mice. Int. Immunopharmacol. 11, 1293–1297. 10.1016/j.intimp.2011.04.011 21530682

[B21] Isoda K. Tanaka A. Fuzimori C. Echigoya M. Taira Y. Taira I. (2020). Toxicity of gold nanoparticles in mice due to nanoparticle/drug interaction induces acute kidney damage. Nanoscale Res. Lett. 15, 141. 10.1186/s11671-020-03371-4 32617798 PMC7332653

[B22] Jander G. Rahme Laurence G. Ausubel Frederick M. (2000). Positive Correlation between virulence of Pseudomonas aeruginosa mutants in mice and insects. J. Bacteriol. 182, 3843–3845. 10.1128/jb.182.13.3843-3845.2000 10851003 PMC94559

[B23] Jia C. J. Schüth F. (2011). Colloidal metal nanoparticles as a component of designed catalyst. Phys. Chem. Chem. Phys. 13, 2457–2487. 10.1039/c0cp02680h 21246127

[B24] Kamoshida G. Kikuchi-Ueda T. Tansho-Nagakawa S. Nakano R. Nakano A. Kikuchi H. (2015). Acinetobacter baumannii escape from neutrophil extracellular traps (NETs). J. Infect. Chemother. 21, 43–49. 10.1016/j.jiac.2014.08.032 25287154

[B25] Kim D. S. Kim Y. T. Hong S. B. Kim J. Huh N. S. Lee M. K. (2016). Development of lateral flow assay based on size-controlled gold nanoparticles for detection of hepatitis B surface antigen. Sensors (Basel) 16, 2154. 10.3390/s16122154 27999291 PMC5191134

[B26] Kohane D. S. (2007). Microparticles and nanoparticles for drug delivery. Biotechnol. Bioeng. 96, 203–209. 10.1002/bit.21301 17191251

[B27] Kouser L. Paudyal B. Kaur A. Stenbeck G. Jones L. A. Abozaid S. M. (2018). Human properdin opsonizes nanoparticles and triggers a potent pro-inflammatory response by macrophages without involving complement activation. Front. Immunol. 9, 131. 10.3389/fimmu.2018.00131 29483907 PMC5816341

[B65] Kristensen N. P. (2003). Handbuch der zoologie = : handbook of zoology. Berlin, NY: de Gruyter.

[B28] Kroll A. Dierker C. Rommel C. Hahn D. Wohlleben W. Schulze-Isfort C. (2011). Cytotoxicity screening of 23 engineered nanomaterials using a test matrix of ten cell lines and three different assays. Part. Fibre Toxicol. 8, 9. 10.1186/1743-8977-8-9 21345205 PMC3059267

[B29] Li L. Jiang L. L. Zeng Y. Liu G. (2013). Toxicity of superparamagnetic iron oxide nanoparticles: research strategies and implications for nanomedicine. Chin. Phys. B 22, 127503. 10.1088/1674-1056/22/12/127503

[B30] Liu Y. Zhu S. Gu Z. Chen C. Zhao Y. (2022). Toxicity of manufactured nanomaterials. Particuology 69, 31–48. 10.1016/j.partic.2021.11.007

[B31] Mabrouk M. Das D. B. Salem Z. A. Beherei H. H. (2021). Nanomaterials for biomedical applications: production, characterisations, recent trends and difficulties. Molecules 26, 1077. 10.3390/molecules26041077 33670668 PMC7922738

[B32] Maslova E. Shi Y. Sjoberg F. Azevedo H. S. Wareham D. W. McCarthy R. R. (2020). An invertebrate burn wound model that recapitulates the hallmarks of burn trauma and infection seen in mammalian models. Front. Microbiol. 11, 998. 10.3389/fmicb.2020.00998 32582051 PMC7283582

[B33] Maslova E. Eisaiankhongi L. Rigole P. Coenye T. McCarthy R. R. (2024). Carbon source competition within the wound microenvironment can significantly influence infection progression. NPJ Biofilms Microbiomes 10, 52. 10.1038/s41522-024-00518-4 38918415 PMC11199515

[B34] Mccarthy R. R. Valentini M. Filloux A. (2017). “Contribution of cyclic di-GMP in the control of type III and type VI secretion in *Pseudomonas aeruginosa* ,” in c-di-GMP signaling: methods and protocols. Editor Sauer K. (New York, NY: Springer New York). 10.1007/978-1-4939-7240-1_17 28889297

[B35] Menard G. Rouillon A. Cattoir V. Donnio P. Y. (2021). Galleria mellonella as a suitable model of bacterial infection: past, present and future. Front. Cell. Infect. Microbiol. 11, 782733. 10.3389/fcimb.2021.782733 35004350 PMC8727906

[B36] Meunier E. Coste A. Olagnier D. Authier H. Lefèvre L. Dardenne C. (2012). Double-walled carbon nanotubes trigger IL-1β release in human monocytes through Nlrp3 inflammasome activation. Nanomedicine 8, 987–995. 10.1016/j.nano.2011.11.004 22100755

[B37] Moore A. Marecos E. Bogdanov A. JR. Weissleder R. (2000). Tumoral distribution of long-circulating dextran-coated iron oxide nanoparticles in a rodent model. Radiology 214, 568–574. 10.1148/radiology.214.2.r00fe19568 10671613

[B38] Moya-Andérico L. Vukomanovic M. Cendra M. D. M. Segura-Feliu M. Gil V. Del Río J. A. (2021). Utility of Galleria mellonella larvae for evaluating nanoparticle toxicology. Chemosphere 266, 129235. 10.1016/j.chemosphere.2020.129235 33316472

[B39] Ngobili T. A. Daniele M. A. (2016). Nanoparticles and direct immunosuppression. Exp. Biol. Med. (Maywood) 241, 1064–1073. 10.1177/1535370216650053 27229901 PMC4950368

[B40] Opris R. V. Baciu A. M. Filip G. A. Florea A. Costache C. (2025). The use of Galleria mellonella in metal nanoparticle development: a systematic review. Chemico-Biological Interact. 415, 111511. 10.1016/j.cbi.2025.111511 40246051

[B41] Palermo M. S. Giordano M. Isturiz M. A. (1991). Effect of cyclophosphamide on the clearance of IgG-sensitized red cells in mice. Clin. Immunol. Immunopathol. 58, 343–351. 10.1016/0090-1229(91)90125-t 1825805

[B42] Park E.-J. Umh H. N. Choi D.-H. Cho M. H. Choi W. Kim S.-W. (2014). Magnetite- and maghemite-induced different toxicity in murine alveolar macrophage cells. Archives Toxicol. 88, 1607–1618. 10.1007/s00204-014-1210-1 24525745

[B43] Patra J. K. Das G. Fraceto L. F. Campos E. V. R. Rodriguez-Torres M. D. P. Acosta-Torres L. S. (2018). Nano based drug delivery systems: recent developments and future prospects. J. Nanobiotechnology 16, 71. 10.1186/s12951-018-0392-8 30231877 PMC6145203

[B44] Peleg Anton Y. Jara S. Monga D. Eliopoulos G. M. Moellering R. C. Mylonakis E. (2009). Galleria mellonella as a model system to study acinetobacter baumannii pathogenesis and therapeutics. Antimicrob. Agents Chemother. 53, 2605–2609. 10.1128/AAC.01533-08 19332683 PMC2687231

[B67] Perdoni F. Falleni M. Tosi D. Cirasola D. Romagnoli S. Braidotti P. (2014). A histological procedure to study fungal infection in the wax moth Galleria mellonella. Eur. J. Histochem. 58, 2428. 25308852 10.4081/ejh.2014.2428 PMC4194400

[B45] Poli G. Schaur R. J. (2000). 4-Hydroxynonenal in the pathomechanisms of oxidative stress. IUBMB Life 50, 315–321. 10.1080/713803726 11327326

[B46] Pondman K. M. Sobik M. Nayak A. Tsolaki A. G. Jäkel A. Flahaut E. (2014). Complement activation by carbon nanotubes and its influence on the phagocytosis and cytokine response by macrophages. Nanomedicine Nanotechnol. Biol. Med. 10, 1287–1299. 10.1016/j.nano.2014.02.010 24607938

[B47] Pondman K. M. Pednekar L. Paudyal B. Tsolaki A. G. Kouser L. Khan H. A. (2015). Innate immune humoral factors, C1q and factor H, with differential pattern recognition properties, alter macrophage response to carbon nanotubes. Nanomedicine 11, 2109–2118. 10.1016/j.nano.2015.06.009 26169151

[B48] Pondman K. Le Gac S. Kishore U. (2023). Nanoparticle-induced immune response: health risk versus treatment opportunity? Immunobiology 228, 152317. 10.1016/j.imbio.2022.152317 36592542

[B49] Prabhu S. Mutalik S. Rai S. Udupa N. Rao B. S. S. (2015). PEGylation of superparamagnetic iron oxide nanoparticle for drug delivery applications with decreased toxicity: an *in vivo* study. J. Nanoparticle Res. 17, 412. 10.1007/s11051-015-3216-x

[B50] Qi Y.-T. Zhang F.-L. Tian S.-Y. Wu H. Q. Zhao Y. Zhang X. W. (2024). Nanosensor detection of reactive oxygen and nitrogen species leakage in frustrated phagocytosis of nanofibres. Nat. Nanotechnol. 19, 524–533. 10.1038/s41565-023-01575-0 38172432

[B51] Schlenk F. Werner S. Rabel M. Jacobs F. Bergemann C. Clement J. H. (2017). Comprehensive analysis of the *in vitro* and ex ovo hemocompatibility of surface engineered iron oxide nanoparticles for biomedical applications. Archives Toxicol. 91, 3271–3286. 10.1007/s00204-017-1968-z 28378120

[B52] Shah A. Dobrovolskaia M. A. (2018). Immunological effects of iron oxide nanoparticles and iron-based complex drug formulations: therapeutic benefits, toxicity, mechanistic insights, and translational considerations. Nanomedicine 14, 977–990. 10.1016/j.nano.2018.01.014 29409836 PMC5899012

[B69] Sheehan G. Clarke G. Kavanagh K. (2018). Characterisation of the cellular and proteomic response of *Galleria mellonella* larvae to the development of invasive aspergillosis. BMC Microbiol. 18, 63. 29954319 10.1186/s12866-018-1208-6 PMC6025711

[B53] Singh N. Jenkins G. J. Asadi R. Doak S. H. (2010). Potential toxicity of superparamagnetic iron oxide nanoparticles (SPION). Nano Rev. 1, 5358. 10.3402/nano.v1i0.5358 22110864 PMC3215220

[B54] Sonavane G. Tomoda K. Makino K. (2008). Biodistribution of colloidal gold nanoparticles after intravenous administration: effect of particle size. Colloids Surfaces B Biointerfaces 66, 274–280. 10.1016/j.colsurfb.2008.07.004 18722754

[B55] Stark W. J. Stoessel P. R. Wohlleben W. Hafner A. (2015). Industrial applications of nanoparticles. Chem. Soc. Rev. 44, 5793–5805. 10.1039/c4cs00362d 25669838

[B56] Stroh A. Zimmer C. Gutzeit C. Jakstadt M. Marschinke F. Jung T. (2004). Iron oxide particles for molecular magnetic resonance imaging cause transient oxidative stress in rat macrophages. Free Radic. Biol. Med. 36, 976–984. 10.1016/j.freeradbiomed.2004.01.016 15059638

[B57] Trevijano-Contador N. Zaragoza O. (2018). Immune response of Galleria mellonella against human fungal pathogens. J. Fungi (Basel) 5, 3. 10.3390/jof5010003 30587801 PMC6463112

[B58] Tsai C. J. Loh J. M. Proft T. (2016). Galleria mellonella infection models for the study of bacterial diseases and for antimicrobial drug testing. Virulence 7, 214–229. 10.1080/21505594.2015.1135289 26730990 PMC4871635

[B59] Vecchio G. Galeone A. Brunetti V. Maiorano G. Sabella S. Cingolani R. (2012). Concentration-dependent, size-independent toxicity of citrate capped AuNPs in *Drosophila melanogaster* . PLoS One 7, e29980. 10.1371/journal.pone.0029980 22238688 PMC3251612

[B60] Wang H. Xu Q. Heng H. Zhao W. Ni H. Chen K. (2024a). High mortality of Acinetobacter baumannii infection is attributed to macrophage-mediated induction of cytokine storm but preventable by naproxen. eBioMedicine 108, 105340. 10.1016/j.ebiom.2024.105340 39303669 PMC11437915

[B61] Wang Y.-L. Lee Y.-H. Chou C.-L. Chang Y. S. Liu W. C. Chiu H. W. (2024b). Oxidative stress and potential effects of metal nanoparticles: a review of biocompatibility and toxicity concerns. Environ. Pollut. 346, 123617. 10.1016/j.envpol.2024.123617 38395133

[B62] Wei H. Hu Y. Wang J. Gao X. Qian X. Tang M. (2021). Superparamagnetic iron oxide nanoparticles: cytotoxicity, metabolism, and cellular behavior in biomedicine applications. Int. J. Nanomedicine 16, 6097–6113. 10.2147/IJN.S321984 34511908 PMC8418330

[B66] Zhang C. Huang P. Bao L. He M. Luo T. Gao G. (2011). Enhancement of gastric cell radiation sensitivity by chitosan-modified gold nanoparticles. J. Nanosci. Nanotechnol. 11, 9528–9535. 22413242 10.1166/jnn.2011.5318

[B63] Ziccheddu G. Proietti E. Moschella F. (2013). The Janus face of cyclophosphamide: a sterile inflammatory response that potentiates cancer immunotherapy. Oncoimmunology 2, e25789. 10.4161/onci.25789 24244905 PMC3825725

